# New Technological Interventions in Conservation Conflicts: Countering Emotions and Contested Knowledge

**DOI:** 10.1007/s10745-017-9936-z

**Published:** 2017-09-11

**Authors:** Audrey Verma, René van der Wal, Anke Fischer

**Affiliations:** 1 Sociology, Claremont Bridge Building, Newcastle University, Newcastle upon Tyne, NE1 7RU UK; 2 Aberdeen Centre for Environmental Sustainability (ACES), University of Aberdeen, School of Biological Sciences, Auris, 23 St. Machar Drive, Aberdeen, AB24 3UU UK; 3 Social, Economic and Geographical Sciences Group, James Hutton Institute, Craigiebuckler, Aberdeen, Scotland AB15 8QH UK

**Keywords:** Knowledge contestation, Digital imaging, Technologies for nature conservation, Evidence production, Maerl, Fal River, Cornwall, UK

## Abstract

New technologies have increasingly featured in environmental conservation conflicts. We examined the deployment of imaging devices such as sonar equipment and cameras to survey the Fal estuary in Cornwall, UK. Due to heavy use of these waters, there have been several disputes coalescing around protected marine features, including the estuary’s rare maerl beds. A comparison of two cases, scallop dredging and docks development, showed technical instruments being deployed to produce information about the marine environment as evidence to inform decision-making. The use of imaging devices stimulated political action and was regarded as a move away from emotion-based decision-making towards desired objectivity. Simultaneously, however, the process of deploying these devices was challenged and there was recognition that the resultant information could be used to construct the estuary as a politically charged space. Thus, rather than clarifying and resolving contentious issues, technological interventions generated new baselines for knowledge contestation and amplified ongoing battles for credibility and authority.

## Introduction

The complex and inherently social dimensions of environmental conservation are more pronounced in conflict situations, where fundamental contestation among the agendas and values of multiple groups of human actors complicates decision-making processes (Marshall *et al.* 2007; White *et al.* 2009; Redpath *et al.* 2015). At the same time, there have been continued calls for ‘evidence-based conservation,’ i.e., the production and analysis of sufficient and robust scientific data to serve as the core knowledge base informing conservation policies (Pullin and Knight 2003; Sutherland *et al.* 2004). New technologies are increasingly employed to expand this knowledge base (Arts *et al.* 2015; August *et al.* 2015). The use of such technologically-produced information in conservation conflicts is located at the complex intersections between the contested, value-laden claims of competing actors and the perceived need for evidence in the form of objective, rational, and politically-neutral scientific knowledge (Ozawa 1996). This raises questions of how such technologies are being deployed, and what broader implications these sorts of interventions might have for the management of environmental conservation conflicts. We address these questions by investigating the narratives revolving around the production and interpretation of information generated by new technologies in the case of maerl conservation in the Fal estuary, Cornwall, UK.

## Conceptual Background

### Information Technologies and Public Participation in Environmental Decision-Making

There is a long-standing debate on the capacity of information-based technologies to enable or hinder public participation in environmental decision-making. It has been noted that the features of these technologies can themselves lead to particular forms of social organisation (Winner 1986; Beck 1992; Marres 2012), for instance by precluding public participation (Fischer 2000). Information technologies have been shown to reproduce normative inequalities (i.e., the digital divide), and be susceptible to issues of centralised control, intrusion into privacy, security risk, and increased surveillance particularly by state and corporate apparatuses. Apart from potentially restricting the quality and quantity of public engagement, these potential ambient harms (Jasanoff 2003) brought about by technocratic solutions (Beck 1992) can also create a general mistrust in technology.

In less explicit ways, the implementation of these technologies has been read as ideological and political. For instance, the use of technologies may be a means of privileging scientific-rational (Lidskog and Sundqvist 2004; Lidskog 2008), ‘neopositivist’ (Fischer 2000), and technological fix perspectives (Huesemann and Huesemann 2011), wherein techno-scientific methods are seen as the only valid modes of eliciting ‘truth’ or achieving ‘progress.’ Further, the deployment of these technologies has been understood as justifying the autonomy of those possessing technocratic expertise and increasing their influence in decision-making processes (Nelkin 1975), which have been shown to result in distinctions between fact and value (Latour 2004), between experience and emotion (Milton 2002; Fazey *et al.* 2006), between objectivity and subjectivity (Duckett *et al.* 2015), between qualitative and quantitative knowledge (Carolan 2006; Adams and Sandbrook 2013), between scientific and traditional/indigenous knowledge (Briggs 2005; Watson-Verran and Turnbull 2005; Nadasdy 2011), and between experts and laypersons (Nelkin 1975; Wynne 1996; Fischer 2000). While these divisions have been critiqued by the authors and others, they have proven durable as social discourses in shaping and defining issues.

At the same time, there are more positive aspects to the relationship between technology and public participation that hold salient implications for environmental decision-making. Mistrust in technologies also necessitates new forms of social organisation, marked by increased public participation and reflexive modernisation (Beck 1992; Gabe 2004). Thus, some of the technologies, such as network-based information and communication platforms that are susceptible to illiberal governance and privileged access, can also have the capacity to heighten awareness and create opportunities for mass participation (Castells 2010) in environmental decision-making (Howes 2002; Zavestoski and Shulman 2002), thereby encouraging ‘environmental citizenship’ (Irwin 1995). Such participatory technologies may arguably facilitate a dwindling of the monopoly of technocratic science on rationality (Hannigan 2006, paraphrasing Beck 1992) since platforms for mass information consumption, dissemination, and reproduction can be means for the public to engage with the perspectives of experts. As Yearley (2005: 94) noted, “the complexity and open-endedness of environmental problems [means] that no single corps of experts [is] likely to be able to claim exhaustive knowledge of any system large enough to be of practical significance. For that reason, lay people who are knowledgeable … of their local environment might be able to act as additional peer reviewers.” Recent drives toward greater expert-public engagement could in turn account for increased integration of local expertise and alternative sources of information with scientific knowledge (Peuhkuri 2002), an increasing hybridity of the identities of actors involved in a given environmental issue (Castree and Braun 1998), and a growing degree of ‘scientisation of protest against science,’ where new and reflexive forms of ‘advocacy science’ emerge to critique traditional and rigid applications of scientific rationality (Beck 1992; Hannigan 2006).

### Complexity, Contestation and Constructionism

Environmental issues and the definition of environmental ‘risks’ (Adam and Loon 2000) have been shown to be more than just matters of fact, being inextricably bound up with the social, and concomitantly, multiple and often conflicting values, agendas and perspectives (Carolan 2006; Marshall *et al.* 2007). Ideas of ‘nature’ are constructed and “fundamentally intertwined with dominant ideas of society […], the project of determining what is a natural impact becomes as much a social and cultural project as it is 'purely' scientific” (Macnaghten and Urry 1998: 15). Given such enmeshment of politics and nature conservation (Carolan 2008), and the observation that conservation is an applied, goal-oriented endeavour (Mace 2014) with value-laden visions of what the nature/society relationship *should* be rather than *is*, it has been suggested that nature conservation may be better conceptualised as a social zone in which scientific and technical knowledge serving regulatory decision-making is produced (Jasanoff 2011), and whereby contested and uncertain knowledge is a key aspect of the process (Funtowicz and Ravetz 1993). Viewed in this manner, rather than being the authoritative or decisive paradigm expected to produce indisputable truths, techno-science becomes one of several available approaches to generate information that may be used to facilitate better understanding, negotiation, and consensus in environmental disputes (Ozawa 1996; Oreskes 2004). Any technologically-derived science-based perspective thus simultaneously becomes more necessary but less sufficient for a definition of truth that is more socially binding (Beck 1992; Peuhkuri 2002).

Within this context, what becomes helpful is not the pursuit of ‘optimal’ solutions as such, but rather an understanding of how problems and solutions come to be defined and are understood by actors. This requires unfolding the normative contexts (Carolan 2008; Juntti *et al.* 2009) within which nature conservation practices and knowledge production take place (Nadasdy 2011). Therefore, rather than weighing up the technical efficacy of technological interventions, we focus on the perceptions and social dynamics emerging from discourses surrounding the techno-scientific production of evidence in order to better understand the varied uses, socio-political underpinnings, and impacts of new devices featuring in environmental conservation conflicts. Given our interest in understanding the relationships coalescing around the deployment of technologies and in the narratives through which actors iteratively assign varied meanings to technologically produced evidence (Hajer 1995; Hannigan 2006), we took a soft constructionist stance and assessed all narratives in a relativist manner, as earning “legitimation and credibility in concrete contexts and in negotiation with other discourses surrounding an issue” (Peuhkuri 2002: 159).

## Methodology

The starting point for our research was an exploratory, qualitative sociological examination of cases where new technologies had been employed to mediate human – nature relationships (Verma *et al.* 2015; Verma *et al.* 2016). The research site and the cases we discuss here came to our attention when we learnt that a diversity of new technologies (including underwater cameras, sonar equipment, and satellite-enabled devices) were being deployed to map the Fal estuary’s seabed features in order to inform ongoing environmental conservation disputes.

### Research Site

**Fig. 1:**
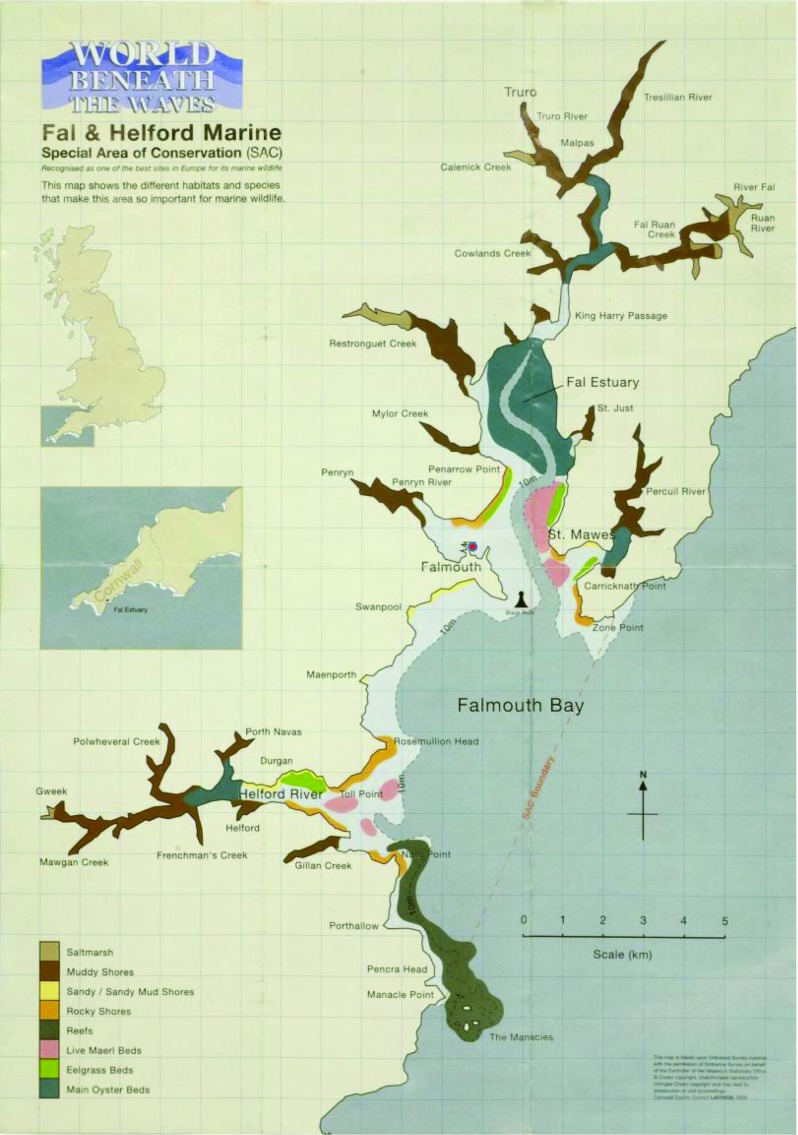
Location of Falmouth, Fal Estuary, and maerl and other sub-features of interest within the Fal Special Area of Conservation (SAC). The SAC boundary is indicated as dashed orange line, primary maerl bed locations as pink polygons, and the approximate location of Falmouth’s port as a red dot (Image source: Cornwall County Council, 2000)

Falmouth, a town on the Fal estuary, is located in Cornwall, a rural county in the southwest of England (Fig. 1). The estuary forms one of the world’s largest natural harbours, which serves as a port, and has been central to the town’s history and its economic fortunes. Given the geography of Falmouth bay and the centrality of the port, there is a plethora of actors with vested interests in the fate of the waters. While legally, as with most coastal waters in the United Kingdom, the waters belong to the Crown, public access rights are enshrined in law.

A large part of the estuary has been classed as a Special Area of Conservation (SAC) (Fig. 1). This classification emerged as a result of the 1994 UK Habitats Regulations, the national enactment of the 1992 EU Habitats Directive. In part due to the difficulties of imposing environmental directives on the marine environment, the focus with identifying SACs was on so-called ‘sub-features’ deemed valuable for protection. During a consultation process, Natural England (formerly English Nature), a scientific advisory body to the government, identified four primary marine features in the Fal estuary that would qualify the habitat for protected status, namely sandbanks, shallow bays, inlets, and reefs. Each of these primary features possesses sub-features of importance: the sandbanks, for instance, were deemed important because of the presence of eelgrass and maerl beds.^1^ This identification resulted in the designation of the Fal and Helford SAC in 1996. An interviewee from the port authorities explained the regulatory implications of this designation:

“[The SAC] covers most of our port limits. And what that designation means is that any development we do, there’s a strict European planning process that we’ve to go through. And this is where we’ve ended with projects costing a lot more money, taking a lot longer in time to put forward and to achieve when it comes to development, predominantly because of that legislation that’s there protecting the environment*.*”

While the designation means that the use of the site is subject to EU law, it is important to note here that the law distinguishes between ongoing activities and new plans or projects: the former calls for management to ensure there is no deterioration of the natural habitat, whereas the latter requires appropriate assessment to be carried out to determine implications for the site in light of conservation objectives before any licenses are granted (Solandt *et al.* 2013). Disputes within SACs are also subject to two principles fundamental to the Habitats Directive: the polluter (or damager) pays principle and the precautionary principle. The first puts the burden of proof that proposed activities would not be damaging on those proposing new developments; the second puts the onus on establishing that proposed activities would not have a negative impact on key sub-features (e.g. maerl) within protected areas. In cases where there is lack of scientific consensus, the precautionary principle requires parties to err on the side of preventive caution.

Maerl is a group of seaweeds containing high levels of calcium and magnesium that give it its crusty, chalky texture and calcified, coral-like appearance, quite unlike other seaweeds. In addition to formation of exoskeletons, maerl grows in dense interlocking mats across the sea floor, producing what looks like a coral structure. Maerl is therefore also known as coralline algae, a form of hard algae (Birkett *et al.* 1998). Maerl requires light for photosynthesis, which limits its presence to shallow waters of up to 20 m in depth (UK Biodiversity Action Plan 2008; Hall-Spencer *et al.* 2010).

While several studies indicate gaps in the present knowledge of maerl beds, even regarding “simple questions such as where the maerl beds occur” (Birkett *et al.* 1998: 10), these studies do suggest that maerl is patchily distributed (Hall-Spencer *et al.* 2010). In the UK, it is rare, and the Fal is one of the few places where extensive live maerl beds are found (SPLASH 2008). It is also for this reason that maerl is a key sub-feature within sandbanks that determined the designation of the Fal estuary as an SAC. Maerl beds are highly biodiverse, serving as a nursery for economically valuable fisheries species (UK Biodiversity Action Plan 2008; see also Seasearch 2012 and McCallum *et al.* 2014). There are several reasons for the patchy distribution of the algae. First, maerl requires rather specific environmental conditions to live and flourish. Second, maerl is slow-growing. Live maerl grows atop dead maerl at the rate of about one millimetre per year and is therefore considered a non-renewable resource (UK Biodiversity Action Plan 2008). Third, due to its free-living and rootless structure, maerl is sensitive to disturbance. Breakages within the mat, even of dead maerl, can weaken the larger maerl bed, making it more prone to erosion (Birkett *et al.* 1998). The recovery potential of maerl is therefore classed as poor (Hall-Spencer *et al.* 2010).

An important feature of maerl, one that became a point of contention in the disputes discussed below, is its unique colour. Described by our interviewees as being “visually stunning” and resembling “purple broccoli sprouting like a carpet across the seabed” often extending further than the eye can see, maerl has a distinct red hue. Dead maerl loses this rich red hue, gradually losing colour through a range of interim colourations, finally turning chalky white.

While maerl as a species has limited legal protection in UK, it is protected as a habitat under the European Commission Habitats Directive 1992 and the UK Biodiversity Action Plan (Birkett *et al.* 1998; Newton 2011). In Cornwall, it is protected as a key sub-feature of a Special Area of Conservation (SAC), as identified under the Habitats Directive (92/43/EEC). Given that the implementation and administration of conservation policies can be especially challenging in marine environments, key sub-features may become focal points for nature conservation policy. This means that the condition of the Fal SAC, for example, is determined in large part by the health and condition of the sub-feature maerl.

### Data Collection

Over the course of 3 weeks in the autumn of 2014, through snowball sampling we interviewed eight key informants over the phone. We were interested in individuals who could respond to questions regarding new technological interventions. However, the (semi-formal) interviews did not focus on the use of technologies in the first instance. Rather, questions regarding technologies were posed in relation to interviewee’s broader understanding of situations (e.g., “…within the context you described what role did sonar imaging play?”). The interviews each lasted between one and two and a half hours. All interviews were recorded with the knowledge and consent of respondents, and were subsequently transcribed and transcripts anonymised. In addition to the transcripts, we also made extensive use of publicly available archival data – feeds and documents from social media sites, radio interviews (transcribed), legal documents, technical reports, and press coverage. While there were multiple conservation disputes revolving around maerl as a protected sub-feature, our interviewees focussed on the most recent conflicts over scallop dredging and planned docks development, the basis of our two case studies.

For the purposes of this paper, we labelled each respondent by their primary self-identification or the capacity in which they granted interviews to us. Our respondents included a technical advisor, a port authority staff member, a scientific campaigner, two fishermen, an advisory group member, and two representatives from (two different) environmental organisations. The scientific campaigner, fishermen, and advisory group member were part of the SAC advisory group, a voluntary community forum described to us by an interviewee as “a means for anybody with an interest and wishes to have a say [about decisions pertaining to the Fal SAC to] get some access to the management forum which is the officially appointed body … that makes the decisions.” While there was little indication of a pro-conservation or pro-development stance being adopted by the advisory group as such, there were clear underlying tensions and antagonisms among the ‘developers’ and individuals who opposed them due to nature conservation and economic reasons. These individuals, including the scientific campaigner and one of the fishermen, were also part of an independent action group organised to protest against docks development plans.

We acknowledge here that the roles played by our respondents overlapped, and while dominant roles did exist for most individuals, understanding their perspectives as attributable to just one role would be too simplistic. For example, most respondents were local residents who had families with histories tied to the marine environment and harbour operations. The fishermen also revealed that they had higher degrees in environmental science-related disciplines. We were thus careful not to overlook the multiplicity of roles and affiliations of our interviewees in our interviews and analysis.

### Data Analysis

Data analysis was conducted by coding data material in NVivo in tandem with further data collection. This parallel process allowed us to iteratively elicit and continually refine concepts of importance (codes). It also allowed us to build a rich description of the complex and heterogeneous social, spatial, and technical contexts of the two disputes. Initial codes were clustered to derive key themes revolving around the deployment of new technologies for supplying data, information, and evidence related to the Fal’s marine environment. Through this data-driven process, we developed interlinked themes to organize our findings, including technical aspects of technology use, social and political aspects of their deployment, limitations, and contestation over the production of data and interpretation of evidence produced.

## Technological Interventions

Anthropogenic activities have been shown to affect maerl beds in the Fal estuary directly and indirectly, for instance with the extraction of maerl for use as fertiliser in agriculture in the past (Birkett *et al.* 1998) and through seabed scouring from the anchors of ships and buoys (Newton 2011). Here, we focus on two recent controversies that form our case studies: the dispute over scallop dredging practices and impacts from docks development. We first provide the background to both cases, and then introduce some of the primary technologies deployed and lay out the technical reasons articulated for such interventions.

The local commercial fisheries industry is centred in the Fal estuary itself, and includes crab potting, lobster and prawn netting, smaller-scale fishing, and a large native oyster farm. Scallop fishing also occurred, and was carried out in two ways: collection by hand (commercial scallop diving) and dredging. Following the designation of the site as an SAC, contention arose particularly with regard to this latter practice, which involved towing heavy metal dredges over protected sub-features of the seabed, including the maerl beds. As a consequence, a by-law was put in place in 2003 to prevent scallop dredging in the estuary,^2^ as the activity was deemed to be damaging to protected conservation features. The activity continued, however, as a consequence of legal vagueness as to whether scallop dredging was an ongoing activity that simply required management to avoid damage and disturbance, or a new plan or project requiring appropriate assessments. Aligned with cultural and economic understandings (i.e., the view that maerl was an abundant resource prone to coming loose naturally), the dredging activity was initially considered as ‘ongoing,’ and presumed to affect mainly maerl that was already dead (Solandt *et al.* 2013). These understandings were disputed by local campaigners and environmental organisations, which in 2006 started the process of challenging the scallop fisheries.

Following their initial petition, a voluntary approach was put in place that allowed scallop dredging for 15 days in each month of November and December in both 2007 and 2008. During this time, calls for complete bans continued whilst assessments were carried out by regulatory bodies. The potential for damage was confirmed by surveys using visual technologies,^3^ namely still/video-cameras that could be dropped and operated under a boat. While such devices may be considered as relatively simple compared to other technologies we would encounter in the docks development case, this technically-aided information-gathering process was crucial in the resulting ministerial order banning all dredging in the Fal in March 2008.^4^

“… in order to see what the habitats were on the seabed, the [fisheries management body] got a boat to survey the seabed and dropped down cameras where they wanted to dredge … and it showed some of the habitat that should be protected. So even the [fisheries management body] couldn’t refute the claim that it was likely to damage the site. … The most important thing was when [the statutory advisory body] said ‘we can’t say it won’t damage it,’ which in legalese ways is saying you have to protect it.” (Environmental organisation 1 staff)

With regard to the development of the Fal’s docks, the harbour was administered as a trust port, meaning that it was managed by a body granted statutory powers by the government. It was, however, governed based on local legislation and controlled by a board of commissioners independent of direction and funding from the government. There were no shareholders or owners as such, and the port was managed for stakeholders (defined as anyone who used the port), with profits being re-invested back into the port. At the time of our study, there were ongoing long-term management plans to widen the channel to accommodate larger ships, which would require dredging the approach channel.

While all our interviewees acknowledged a generally high level of support from local residents for the development plans, environmental and natural resource management concerns formed the basis for a vocal and active opposition to the proposed dredging to widen the channel. In addition to the direct impact on seabed features that would be in the dredge path, the plans raised concerns from fishermen and environmentalists. They believed that the resultant sedimentation within the water column over the protracted proposed dredging period would negatively affect protected features and biodiversity resources within the spoil grounds, i.e., those parts of the marine environment where the dredge material would be deposited.

As a proposed plan, environmental assessments were required as part of the application process. In order to establish the potential impact on the maerl beds, three interlinked issues had to be addressed. First, the extent of maerl: how much there was and where exactly it was located*.* Partly due to the unique challenges posed by the marine environment, the considerable spatial scale involved and financial constraints of regular surveying of the estuary, one of our interviewees, a staff member of an environmental organisation, explained that there had been no sufficiently extensive or regular mapping of maerl, although there was a general idea of what was in the estuary from diver surveys carried out during the identification of the site as an SAC (pre-designation).

Second, the issue of how to tell whether maerl is dead or alive centred on two closely related characteristics – mobility and colour. With regard to mobility, since fragments of maerl can break off and move to new locations with currents, the classification of individual branches and the mapping of viable (live) maerl bed boundaries are difficult. This is further complicated by the fact that colour is used to determine whether maerl is dead or alive. Maerl’s colour changes gradually - from red/purple to orange, grey, brown and white - as a branch dies (see above).

To overcome the issue of scale and to make visible the otherwise ‘unseeable’ in marine environments, several types of technological interventions were deployed.^5^ Bathymetric sonar was used to produce a 3-D model of the seabed (to closely approximate depths at different locations), and side-scan sonar was used to give assess the structure of the seabed. Drop-cameras were deployed to ground-truth the sonar-based seabed model and structure. These cameras were paired with Global Positioning Satellite (GPS) devices and Geographic information system (GIS) software, to respectively determine location coordinates where each image was taken and to spatially map maerl locations and extent:

“The difficulty we have is that to see the unseeable, in other words, what’s down at the bottom of the sea. … We need technology to be able to see things we can’t do with our eyes. Now, the simplest solution might be to try and get a camera in there. Very often it’s a matter of using remote sensing technology like for instance sonar in the water or magnetic or electrical methods underground.” (Technical advisor)

These sets of technological surveying methods provided the baseline information required for mandatory planning applications. They were also employed to address a third issue, namely whether relocation of maerl beds would be a viable strategy, i.e., whether relocated fragments would survive and develop into thriving beds. Further deployment of technologies during maerl relocation trials were regarded by some actors as enabling high-precision sampling:

“[What the technical advisor did] was really valuable [in selecting sites] for the maerl relocation trials. … What we did [for the relocation trials was] we used RTK [Real Time Kinematic] …. Essentially it hooks up with a satellite so it gets positioning and we ended up with a trial with accuracy where we knew exactly where we put the dredger on the seabed … within 2cm, which is incredibly accurate when you think of all the different variables that are working on you as you’ve got something underwater. … Lines were straight lines. They weren’t dodgy lines. … we wouldn’t have been able to do the trial dredge without those technologies being available.” (Port authority staff)^6^

Here, therefore, the technologies were deployed within the marine environment to visualise and survey otherwise obscured terrain and to provide what was represented as precise and accurate information in relation to the location, extent, and condition of a protected sub-feature such as maerl beds. These heavily technical processes produced data that the port authorities submitted to the state regulatory agency as part of the body of evidence required for approval of a planning application.

## Techno-Political Interventions

In the scallop dredging case, when in 2006 a key player initiated action in the Fal, there was a sense that the committees and regulatory bodies in charge of protecting the SAC were refusing to engage with the concerns of local protestors. In addition to legal vagueness and cultural perceptions, there was a belief that those in positions of power to interpret and implement policies were lacking in political will:

“[The bureaucracy] have a well-worn set of tactics that they use for avoiding having to deal with people like me. They just ignore you or they don’t answer your e-mails, or they give you the run-around and hope that you just get bored. You just have to keep on engaging with them … and frankly embarrassing them and making it impossible for them to hold these flawed positions. … What you are battling is political will. The political will to just do the right thing isn’t there*.*” (Scientific campaigner)

To this end, the scientific campaigner allied with non-government organisations in efforts to engage the relevant bodies. More than strategic alliances and persistent protest, it quickly emerged that visual technological interventions were key in providing effective ‘embarrassment’ that stimulated political action. Photographs taken of scallop dredgers operating in the area were submitted as evidence of illegal activity, and these images provoked engagement from relevant bodies that had previously ignored campaigners. Further, our informants believed that knowledge of European legislation coupled with the precautionary principle and secondary scientific data showing the effects of scallop dredging on maerl beds impressed upon the regulatory bodies the severity of protestors’ concerns, and highlighted urgent cause for action:

“Ideally, you would have everything, but what we do lack is the photos, in most instances. … What [the scientific campaigner] did in order to stimulate this whole thing was to simply take a photo of a scallop dredger with a known headland in the background. ... He used a high-res camera and you could see … the metal ropes which go down to the dredgers on the seabed. You can see them restrained so the gear was being towed. So by taking such very important evidential documentation of activity, you can’t refute it … The guys were, I think we call it, bang to rights. … All we had to do is just go into a room and say [to the regulating bodies], 'here’s a photo of a boat that is scallop dredging, here’s the scientific paper from 1996 on the effects of scallop dredging on maerl' … Put the two and two together with something called the precautionary principle. ….” (Environmental organisation 1 staff)

The submission of consolidated complaints prompted a course of action by regulatory bodies, leading to the banning of scallop dredging in the Fal. Subsequent studies by the fisheries management body confirmed that the practice was likely to be damaging and resulted in the eventual ban. This outcome in turn motivated another lobbying attempt by the scientific campaigner and environmental organisation (alongside a law firm) in 2012, which led to the government implementing proactive management measures to safeguard marine SACs from potentially damaging activities.

### Technologically-Produced Evidence as a Counter to Emotional Arguments

The theme of countering emotive arguments with ‘hard evidence’ (equated with ‘scientific data’) emerged as salient in our interviews. It was not just actors in support of development or scientists who believed that such data were needed. All actors we interviewed emphasised the need for evidential data. For instance, a senior committee member of the advisory group generally took a neutral stance in the docks development debate, but argued that there was marked need for fewer feelings and more knowledge and data by way of evidence. Another respondent, a fisherman, emphasised the need for reasoned, sensible, rational, and objective arguments rather than emotional (or emotive), liturgical, or extreme views. However, all of our interviewees articulated that opposing actors (and indeed even some allies) were guilty of emotionality or irrationality. In this sense, environmentalists were just as likely as fishermen, developers, or scientists to articulate views implying that other actors appeared lacking in balance, common sense, reason, and objectivity.

Within the context of such understandings, new technologies were seen as able to produce ‘hard evidence.’ For some actors, these devices became the conduit through which the emotive arguments of others could be countered. When queried about the role of technology for environmental conservation purposes, one interviewee, who had carried out some of the technical surveying work with sonar and ground-truthing methods to determine maerl baselines, stated:

“The problem with conservation that I’ve encountered is that an awful lot of it is driven by well-meaning people, but based purely on emotive arguments. But to do anything effectively, you really need to base it on data. ... the difficulty is in understanding exactly what that habitat is, where it is, and what affects it. ... What tends to happen is that the debate becomes very emotive, and extreme. ... the work that I’m doing is attempting to show that actually a lot of what they’re saying on the emotive side is wrong. That doesn’t mean to say that we shouldn’t be doing something about conservation. But what I’m arguing is that we should have a more scientific, data-driven approach*.*” (Technical advisor)

In the case of docks developments, technological data production was part of the legal requirements for planning applications. However, the measures were also clearly carried out to provide scientific data to address and counter the concerns of local groups vocally opposed to the dredging for development of the docks:

“People are very emotional, very emotive about the sea. … People sort of have a feel, like it’s the common good I suppose, when it comes to water. So although it’s our seabed - we own the seabed and we regulate the water that is above it - we have had a few campaigners involved … just fundamentally opposed to the dredging happening and it’s been quite a challenge …. We’ve had to go away and make sure we have the science to be able to give to them, so it’s made this project probably one of the best projects for scientific data. Every question you’ve got, we’ve got some data about it somewhere.” (Port authority staff)

### ‘Not a World of Red and White’

While all actors we spoke to agreed that ‘hard evidence’ was necessary to manage the Fal and Helford SAC and its protected sub-features, and some actors viewed new technologies as a way to produce evidence that would counter the perceived emotionality and dogmatism presumed to lead to bad management decisions, there was still no overall consensus of what was ‘scientific’ and constituted valid knowledge. These disagreements over the production of information and interpretation of data coalesced around the use of the technological devices. In terms of the production of knowledge, we noted above that technological interventions were seen by some actors as providing precision and accuracy in mapping the baseline conditions of protected sub-features and of giving rich insights into otherwise ‘unseeable’ terrain. However, others highlighted the limitations of these techno-scientific setups and raised the need for a combination of technologies, including less technologically-reliant but, in their understanding, more comprehensive methods of acquiring data (for example, diver surveys):

“I’m quite prepared to believe that an acoustic technology can differentiate maerl from say rock or sand, but there is no way on earth it can detect the difference between live maerl and dead maerl and obviously that’s a really critical issue. So these things are useful but like every methodology in science they have their limitations.” (Scientific campaigner)

We noted above most of our interviewees had a background in a natural science discipline and the consensus across the range of informants regarding the need for evidence and data. This, however, did not lead to consensus over what was ‘scientific.’ Rather than opposition between ‘local’ and ‘scientific’ knowledge, or fundamental differences between ‘expert’ and ‘non-expert’ perspectives, issues arose over who had the right means to derive evidence. A senior advisory group member explained that most people on the advisory group were there as volunteers with day jobs, so did not have the time and resources needed to form “definitive [and] evidential-based” opinions. He contrasted the advisory group to people who were being paid to conduct research. There were also issues in defining who had integrity and therefore objectivity. For example, a fisherman interviewee questioned the funding sources of some of the third party groups commissioned to do research for the developers. Further, there was contestation over who was sufficiently qualified to produce credible information and interpret data reliably.

Much contestation concerned existing interpretations and representations of technologically-produced data, for example that the findings emerging from the maerl relocation trials (using Real Time Kinematic technologies) could not be extrapolated to the scale of the planned development dredging:

“… the disturbance they created in the [maerl relocation] trial is very different to what will happen in the full scale dredge. So it’s simply impossible to extrapolate from the trial to the full scale dredge. You know, it’s like looking at recovery from a paper cut and trying to … assert that if you can recover from a paper cut in two weeks, then you’d recover … from open heart surgery in two weeks.” (Scientific campaigner)^7^

In terms of establishing baseline conditions of protected sub-features in the estuary, the colour of maerl remained a tricky issue, and limitations were acknowledged. One respondent discussed colour fidelity, which is affected by factors such as distance from maerl bed and position or angle relative to the observer, and also the effects of water depth and lighting conditions on how the colour of the maerl was interpreted. For instance, in good light, the reddish-purple colour of the maerl shows well. However, red light is readily absorbed in water, and the deeper the maerl, the more the colour captured by images will be distorted, with important consequences for classification of beds and branches of maerl as dead or alive:

“…the data being presented does not distinguish between what is clearly a viable maerl bed and … just fragments washed somewhere else. There’s no distinction whereas what I’d like to do is actually have three categorisations – a dead maerl bed, a live maerl bed, and an area of largely live maerl fragments to actually show that you get this graduation. It’s not a world of black and white, or red and white in this case.” (Technical advisor)

Such ambiguities with regard to the colour, status and viability of maerl, persistent despite technological interventions, had larger implications for the dispute over the extent and locations of the protected feature. This contributed partly to charges of misrepresentation of data, providing ‘misleading information’ and ‘spinning the science.’

We were given two perfectly opposed sides to the same issue, both using the same sorts of visual technological surveying techniques and involving the same regulatory body. It is also interesting to note the different forms of knowing each side relied on. Where the campaigner emphasised his first-hand experience and scientific knowledge that went alongside video surveys, the advisor underscored the more technical aspects of distinguishing and plotting viable maerl colonies:

“What happened is that the proponents of the dredging … basically try to pretend they missed … when they do the survey of the area, they try to pretend there was virtually no live maerl there, that there was so little that it could effectively be ignored. Well, that was completely untrue. …I actually got hold of their survey reports and raw data and was able to show that … this was a misrepresentation. They basically spun their own science. … I’ve done a lot of diving in that area and I know, I’ve seen the sea bed there. I know there’s lots of live maerl down there. And … I took some video … it wasn’t formal survey work, but I took some underwater video and I sent the co-ordinates to the [regulatory body] and said, ‘… you’re being had here, you’re being misled’*.*” (Scientific campaigner)

“[the port authority] went into a meeting, and [the regulatory body] were talking about the live maerl bed immediately outside the docks. And [the port authority] played them one of the videos to show that actually this alleged live maerl bed was in fact dead. So again, even [the regulatory body] are being given misleading information. … What a lot of previous work used by the activists [has] done, is they’ve used diver surveys, and wherever a diver finds a piece of maerl that’s still pink, they clock that as a live maerl bed, even if it’s washed off and might be a kilometre away. Technically, it’s not yet dead, but it’s not a viable piece of maerl. It is going to die. It’s no longer in a place it can live. So what the activists have is … a lot of areas plotted as live maerl which actually, technically, you could argue, it’s not yet dead but it’s not actually a thriving, viable maerl colony.” (Technical advisor)

## Discussion and Conclusions

Our research revealed a landscape of considerable complexity, where technologically-mediated knowledge production and interpretation were intrinsically bound up with the political, so that the use of evidence-producing devices was simultaneously effectual and problematic in managing the course of conflicts.

In one sense, visual technological devices played a persuasive communicative role in the push for the policy outcome of banning scallop dredging. These technologies were regarded by local activists and environmental organisations as having produced key evidence that stimulated action from what they felt were non-responsive regulatory bodies. In some instances, therefore, our findings showed that visual technologies can have mobilising effects when employed by members of the public pursuing environmental justice (Cohen and Ottinger 2011), highlighting the strategic potential of technological devices to motivate ‘political will’ and draw wider attention to otherwise localised environmental conflicts. This potential is particularly salient given that the technologies we focussed on in the scallop dredging case (digital still cameras and video cameras) are not particularly exclusionary in terms of affordability (at least within the context of our research site), and have indeed become increasingly accessible to the general public in recent years. However, it is important to underscore that the use of these devices in our study did not stand alone in facilitating ‘environmental democracy’ (Jasanoff 1996), but was part of a multi-pronged approach including in-depth understanding of legislation, working alliances with established groups, and reference to recognised scientific data. At a broader level, this implies the need for members of the public to be informed of institutional and legal processes in environmental disputes, and to be given open access to scientific data, or at least to be part of larger networks that provide integral support in order for their technologically-aided protest actions to be effectual.

In another sense, it was clear that the technologies were utilised to provide technical information (e.g., about the baseline conditions of maerl) that would serve as evidence for legally-required assessments. Here, therefore, technologies such as sonar, GPS, and RTK devices were deployed by some actors to produce precise, accurate, and valuable data under challenging marine conditions, and were thus an important means of addressing the ‘evidence trap’ situation documented by Cook *et al.* (2013), where lack of evidence of impact is perceived as evidence that there is no impact.

However, it also emerged that these techno-scientific procedures were entrenched in the political. Most prominently, technologies were used by some actors as a means of producing evidence to counter ‘emotional’ arguments. Interestingly, in our case studies, the traditional oppositions between scientific and lay knowledge did not occur: All of our interviewees, independent of their current occupation, highlighted their educational backgrounds in natural science disciplines and aligned on the need for scientific data and evidence. This did not, however, result in consensus over who or what was rational, balanced, and reasonable rather than emotional. This aspect of our findings brings to the fore a lesser examined aspect of environmental conservation conflicts, raising questions of why emotions are considered a liability when negotiating conservation and how the devaluing of emotions is employed as a strategy in undermining competing claims (Milton 2002).

Relatedly, it became apparent that the debate, while dependent on ‘factual’ rhetoric drawing on scientific and technical knowledge, simultaneously saw technology and science become platforms for opposing claims (Peuhkuri 2002). This meant that actors recognised that technologically-derived representations could serve to provide evidence and to gain credibility in decision-making processes. Even where new technologies were generally regarded as a step towards desired objectivity away from emotion-based decision-making, some actors simultaneously contested the techno-scientific knowledge production and data interpretation processes. For instance, in the docks development case, in addition to actors highlighting the limitations of information-producing technology such as sonar imaging, contention occurred on grounds of the integrity and methodological rigour of those using the devices. Further, some actors articulated concerns that the new technologies effectively excluded particularly members of the public who did not possess the resources needed to produce primary data and lacked the time to analyse the information derived from these devices. This also ties in to existing debates on public participation (or exclusion) from environmental decision-making processes and the role of technologically-derived knowledge in these situations (Fischer 2000). More importantly, the issue underlying the concerns articulated by some of our respondents was that the use of new devices represented a shift in baselines in the battle for credibility and authority. These concerns raise further questions of whether the use of ever-newer instruments are part of a runaway process in which new techniques simply generate new points of technical contention, and if this process serves to reduce or re-establish gaps between ‘expert’ and ‘layperson’ and among different scientific disciplines.

From another perspective, the contests over technologically-derived information also implied a perception that using these devices entailed performative aspects, given that actors recognised that the technologies were not simply apparatuses to produce knowledge or to persuade, but also means to produce representations that could be deployed to materially and discursively construct the estuary as a politically charged space (Rose-Redwood and Glass 2014). This was exemplified in the disagreements over the locations of viable maerl beds, related to conceptual difficulty in defining whether the maerl was alive, dead, or dying. Underwater footage served as material evidence that made the maerl visible and tangible, proving or disproving its presence or absence in particular locations. Even if the interpretation of such footage (i.e., the colour and therefore the condition of the maerl) was contested, being able to produce such footage allowed actors to support and reiterate their respective arguments, or at least respond to contradictory arguments on their own terms. Using technological devices was thus more than about increasing knowledge: resulting visual images enabled actors to articulate their respective arguments in order to make an impact and take action (Marres 2012). It is also worth noting here that the maerl was itself resistant to technological surveillance, and that many of the techno-scientific methods could not guarantee straightforward and unambiguous visual representations that would not be open to conflicting interpretations. This draws attention to the role of the maerl beds and technological devices themselves in defining the conservation issues and generating public participation in the Fal estuary (Callon 1986; Marres 2012).

Our findings show that the use of new technological devices in disputed environmental conservation situations can be both complex and ambiguous. While these instruments can produce data that may be used to motivate resolutions and to produce necessary evidence in some environmental conservation disputes, they may also exacerbate the “problematic aspects of attempting to employ scientific expertise in areas of public concern” (Yearley 1996: 187) or indeed generate new forms of contestation, e.g., over the interpretation of new visual information. We therefore suggest that if the shared eventual aim of actors is to productively manage environmental disputes, then “high-quality technical analysis [and] scientific knowledge has to be produced in tandem with social legitimation” yielded from processes of trust-building through community-based institutions (Jasanoff 1996). This is because the production of data by ever-newer technologies in isolation does not appear to address underlying socio-political disagreements. Rather, existing tensions may be intensified by increasingly passionate disputes between different groups of actors over more and more technical details of conservation-related measures, thus overlooking the broader contexts and relationships within which the disputes occur.

## Footnotes

1
http://jncc.defra.gov.uk/protectedsites/sacselection/sac.asp?EUCode=UK0013112

2
http://www.uklaws.org/statutory/instruments_32/doc32303.htm

3
http://www.seas-at-risk.org/images/pdf/archive/Fal_and_Helford_synopsis-1.pdf

4
http://www.legislation.gov.uk/uksi/2008/2360/pdfs/uksiem_20082360_en.pdf

5
http://stephburnsfameproject.weebly.com/journal-article.html

6 See also https://www.gov.uk/government/uploads/system/uploads/attachment_data/file/332240/1401-maerltrialreport.pdf

7 See also https://www.gov.uk/government/uploads/system/uploads/attachment_data/file/332244/140303-isapreport.pdf
